# The Genomic Landscape of the Fungus-Specific SWI/SNF Complex Subunit, Snf6, in *Candida albicans*

**DOI:** 10.1128/mSphere.00497-17

**Published:** 2017-11-15

**Authors:** Faiza Tebbji, Yaolin Chen, Adnane Sellam, Malcolm Whiteway

**Affiliations:** a Infectious Diseases Research Centre-CRI, CHU de Québec Research Center (CHUQ), University Laval, Quebec City, Quebec, Canada; b Department of Biology, McGill University, Montreal, Quebec, Canada; c Department of Biology, Concordia University, Montreal, Quebec, Canada; d Department of Microbiology, Infectious Disease and Immunology, Faculty of Medicine, University Laval, Quebec City, Quebec, Canada; Carnegie Mellon University

**Keywords:** *Candida albicans*, SWI/SNF complex, carbon utilization, genomic occupancy, morphogenesis

## Abstract

SWI/SNF is an ATP-dependent chromatin-remodeling complex that is required for the regulation of gene expression in eukaryotes. While most of the fungal SWI/SNF components are evolutionarily conserved with those of the metazoan SWI/SNF, subunits such as Snf6 are specific to certain fungi and thus represent potential antifungal targets. We have characterized the role of the Snf6 protein in *Candida albicans*. Our data showed that although there was low conservation of its protein sequence with other fungal orthologs, Snf6 was copurified with bona fide SWI/SNF complex subunits. The role of Snf6 in *C. albicans* was investigated by determining its genome-wide occupancy using chromatin immunoprecipitation coupled to tiling arrays in addition to transcriptional profiling of the *snf6* conditional mutant. Snf6 directs targets that were enriched in functions related to carbohydrate and amino acid metabolic circuits, to cellular transport, and to heat stress responses. Under hypha-promoting conditions, Snf6 expanded its set of targets to include promoters of genes related to respiration, ribosome biogenesis, mating, and vesicle transport. In accordance with the genomic occupancy data, an *snf6* doxycycline-repressible mutant exhibited growth defects in response to heat stress and also when grown in the presence of different fermentable and nonfermentable carbon sources. Snf6 was also required to differentiate invasive hyphae in response to different cues. This study represents the first comprehensive characterization, at the genomic level, of the role of SWI/SNF in the pathogenic yeast *C. albicans* and uncovers functions that are essential for fungal morphogenesis and metabolic flexibility.

**IMPORTANCE** *Candida albicans* is a natural component of the human microbiota but also an opportunistic pathogen that causes life-threatening infections in immunosuppressed patients. Current therapeutics include a limited number of molecules that suffer from limitations, including growing clinical resistance and toxicity. New molecules are being clinically investigated; however, the majority of these potential antifungals target the same processes as do the standard antifungals and might confront the same problems of toxicity and loss of efficiency due to the common resistance mechanisms. Here, we characterized the role of Snf6, a fungus-specific subunit of the chromatin-remodeling complex SWI/SNF. Our genomic and phenotypic data demonstrated a central role of Snf6 in biological processes that are critical for a fungal pathogen to colonize its host and cause disease, suggesting Snf6 as a possible antifungal target.

## Abstract (precis)

*Candida albicans* is a natural component of the human microbiota but also an opportunistic pathogen that causes life-threatening infections in immunosuppressed patients. Current therapeutics include a limited number of molecules that suffer from limitations, including growing clinical resistance and toxicity. New molecules are being clinically investigated; however, the majority of these potential antifungals target the same processes as do the standard antifungals and might confront the same problems of toxicity and loss of efficiency due to the common resistance mechanisms. Here, we characterized the role of Snf6, a fungus-specific subunit of the chromatin-remodeling complex SWI/SNF. Our genomic and phenotypic data demonstrated a central role of Snf6 in biological processes that are critical for a fungal pathogen to colonize its host and cause disease, suggesting Snf6 as a possible antifungal target.

## INTRODUCTION

*Candida albicans* is a normal commensal of humans that can become a potentially life-threatening fungal pathogen in immunosuppressed patients. Systemic infections resulting from *C. albicans* are associated with mortality rates of 50% or greater despite currently available antifungal therapies (1–3). There are presently a limited number of antifungal molecules and drug targets due to the eukaryotic nature of fungi, which makes them similar to their hosts and complicates the targeting of processes that are intrinsically associated with the fungus, and also due to rising cases of resistance. Currently, many new molecules are being preclinically or clinically investigated; however, the majority of those potential antifungals target the same processes as do the standard antifungals and would likely confront the same problems of toxicity and loss of efficiency due to the common resistance mechanisms (4). Hence, an antifungal agent with a new mechanism of action would be advantageous for treatment of fungal infections.

SWI/SNF is an ATP-dependent chromatin-remodeling complex required for the regulation of gene expression in eukaryotes (5). Through its catalytic subunit Snf2, SWI/SNF promotes ATP-dependent nucleosome repositioning by sliding nucleosomes on DNA or evicting histones to allow the binding of transcription factors to their promoters (5). In the yeast *Saccharomyces cerevisiae*, SWI/SNF sliding/eviction activity was found to be required for the binding of different transcriptional regulators, such as Gal4, Hap4, and Gcn4, to allow activation of their target genes (6, 7). The yeast SWI/SNF controls the mRNA levels of a limited set of genes (2 to 5% of all yeast genes), suggesting a specific functional regulation (8). Recent studies have shown that the SWI/SNF complex exhibits structural modularity (subcomplexes) that can differentially regulate the expression of different sets of genes (9, 10). For instance, under the same growth conditions, the transcript levels of genes for ribosome biogenesis and rRNA processing in *S. cerevisiae* were downregulated in an *snf2* mutant strain but not in an *snf5* strain. Similarly, transcripts of sulfur metabolism and glycolysis were upregulated in the *snf2* but not in the *snf5* strain. These results suggest that even if Snf2 and Snf5 are components of the same complex, they have a differential contribution to gene expression control (9).

In *C. albicans*, both Snf2 and Swi1 subunits were shown to be essential for proper differentiation of invasive hyphae as well as virulence in a mouse model of systemic infection (11). Recently, the SWI/SNF complex was found to control the expression of the major facilitator transporter Mdr1, which mediates azole resistance in *C. albicans*. Occupancy of the transcriptional activator Mrr1 to the promoter of the Mdr1 transporter gene was completely dependent on the nucleosome sliding activity of Snf2 (12). Interestingly, genetic inactivation of *SNF2* in azole-resistant strains with an *MRR1* gain-of-function mutation significantly sensitizes the cells to fluconazole (12). Taken together, small molecules targeting this complex could provide a dual action by both abolishing *C. albicans* virulence and restoring azole sensitivity to resistant strains. However, given the conservation of this chromatin-remodeling complex in humans, inhibitors of the *C. albicans* SWI/SNF might also alter the function of its host counterpart.

In addition to the catalytic subunit, Snf2, SWI/SNF is made up of 11 subunits in yeast and *C. albicans* (13, 14). While most members of the fungal SWI/SNF complex are conserved with the metazoan SWI/SNF, subunits including Snf6, Snf11, Taf14, and Swp82 are specific to fungi (13). Since SWI/SNF is a potential antifungal target, chemical perturbation of specific fungal subunits would be a good approach to avoid cross-interaction with human SWI/SNF activity. Here, we have focused our investigation on the characterization of the role of the fungus-specific SWI/SNF subunit, Snf6. Our data show that, although the *C. albicans* subunit has only limited sequence similarity to other fungal orthologs, Snf6 was copurified with SWI/SNF complex subunits including the catalytic ATPase subunit, Snf2. We show that Snf6 plays a critical role in biological processes that are essential for fungal pathogenesis, including carbon metabolic flexibility, stress response, and morphogenesis. The Snf6 regulon was determined by combining both genome-wide location (chromatin immunoprecipitation with microarray technology [ChIP-chip]) and transcriptional profiling (microarrays) to identify targets of the SWI/SNF complex under both yeast- and hypha-promoting conditions.

## RESULTS

### The ORF C2_03930C_A encodes the SWI/SNF fungus-specific subunit, Snf6.

**FIG 1:**
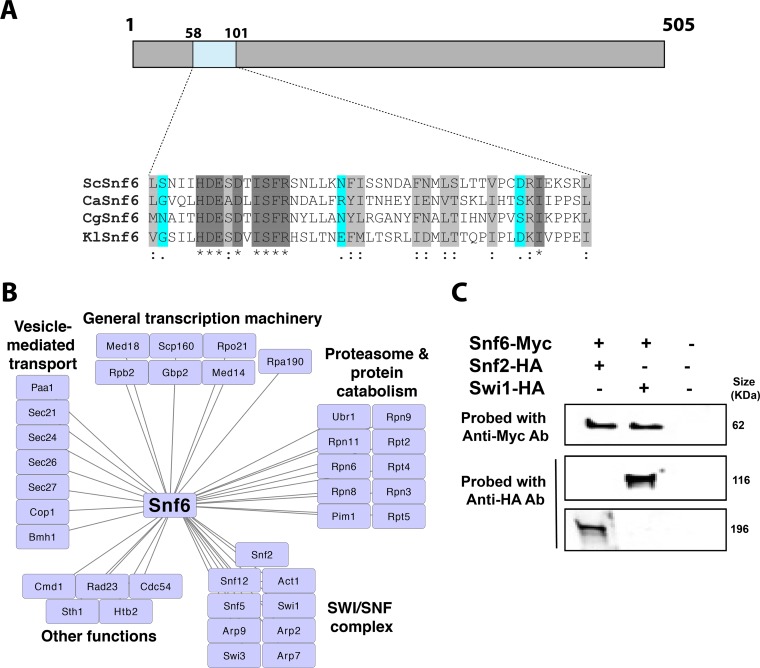
*C. albicans* ORF C2_03930C_A encodes the SWI/SNF fungus-specific subunit, Snf6. (A) Conserved N-terminal region of fungal Snf6. The similarity of the conserved CaSnf6 regions to ScSnf6 (*S. cerevisiae*), CgSnf6 (*C. glabrata*), and KlSnf6 (*Kluyveromyces lactis*) is shown. Identical residues are indicated with asterisks. Conserved and semiconserved substitutions are denoted by colons and periods, respectively. (B and C) Snf6 is a bona fide component of the SWI/SNF complex. Both tandem affinity purification–mass spectrometry (B) and coimmunoprecipitation (C) confirmed the interaction of Snf6 with the SWI/SNF complex. Ab, antibody.

We used *Candida glabrata* Snf6 (CgSnf6) (15) as a query to identify the *C. albicans* Snf6 homolog using BLAST analysis. Our search returned the open reading frame (ORF) C2_03930C_A as a sole hit with 35% identity to CgSnf6. With the exception of a small N-terminal region (positions 58 to 101), the Snf6 protein sequence was not well conserved among the ascomycetes (Fig. 1A). Further, using the CGD alignment function, we looked for the orthologs of the ORF C2_03930C_A in the *Candida* clade and got a set of alignments that identified the region that was most similar among the species.

To confirm that C2_03930C_A is the bona fide *C. albicans* Snf6 (CaSnf6), we used tandem affinity purification (TAP) to comprehensively characterize the Snf6-containing protein complex. Snf6 complexes were affinity purified from log-phase *C. albicans* cultures growing in rich medium (yeast extract-peptone-dextrose [YPD]) using a TAP-tagged Snf6 subunit, and protein interactors were identified using mass spectrometry (MS) from in-solution-digested trichloroacetic acid (TCA)-precipitated proteins or SDS-PAGE gel bands. In total, 116 proteins were identified, including 9 proteins that are known SWI/SNF complex subunits (Fig. 1B; also see Table S1 in the supplemental material). This includes the ATPase catalytic subunit, Snf2, in addition to Snf5, Snf12, Swi1, Swi3, Arp2, Arp7, Arp9, and Act1. In accordance with its role in transcriptional control, Snf6 also copurified with proteins related to general transcriptional machinery, such as RNA polymerases I (Rpa190) and II (Rpo21 and Rpb2), the mediator complex (subunits Med14 and Med18), and the core histone protein, Htb2. Interestingly, Snf6 interacted with the Snf2 homolog, Sth1, which is the catalytic subunit of the essential RSC (Remodel the Structure of Chromatin) complex. This suggests that both SWI/SNF and RSC chromatin remodelers may cooperate to modulate gene expression in *C. albicans*. Snf6 interactors were also enriched in functions related to the proteasome and protein catabolism in addition to vesicle trafficking (Fig. 1B). Coimmunoprecipitation was used to confirm the interaction of Snf6 with two SWI/SNF subunits, Snf2 and Swi1 (Fig. 1C).

10.1128/mSphere.00497-17.1 TABLE S1  List of *C. albicans* Snf6-interacting proteins using TAP procedure. Download TABLE S1, XLSX file, 0.1 MB. Copyright © 2017 Tebbji et al. 2017 Tebbji et al. This content is distributed under the terms of the Creative Commons Attribution 4.0 International license.

### Genome-wide occupancy of Snf6.

**FIG 2:**
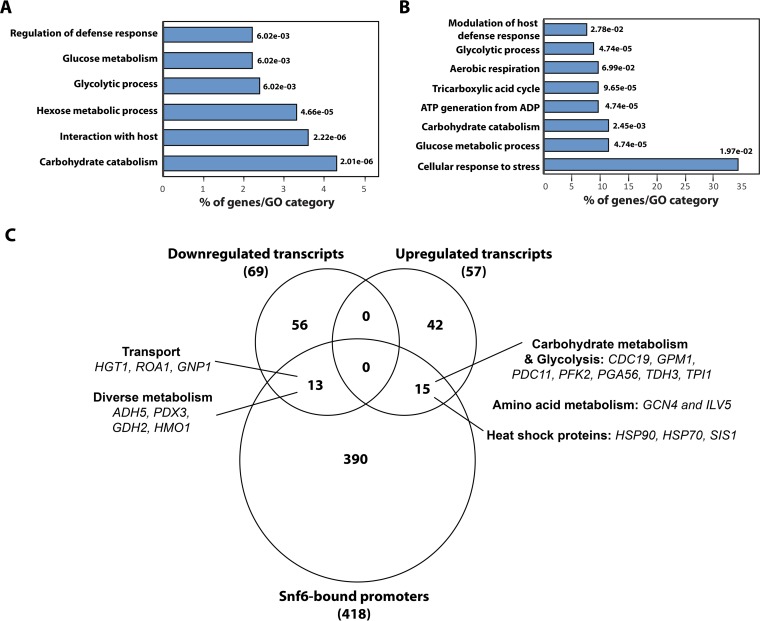
Genome-wide location of Snf6 and transcriptional profiling of *snf6* mutant. (A) Gene ontology of biological process associated with Snf6-bound promoters. The *P* values were calculated using hypergeometric distribution as described in the GO Term Finder Tool website (http://candidagenome.org/cgi-bin/GO/goTermFinder). (B) Gene ontology analysis of upregulated transcripts of *snf6*/Tet-*SNF6* mutant under repressible conditions. Cells were grown in YPD in the presence or absence of 40 µg/ml doxycycline for 48 h and analyzed for gene expression profiles by DNA microarrays. Snf6-dependent transcripts were obtained by comparing the transcriptome of mutant cells treated with doxycycline to that of nontreated cells and by applying a 1.5-fold change cutoff and Welch’s *t* test with a false discovery rate of less than 5%. (C) Venn diagram showing overlaps among genes differentially regulated in the *snf6*/Tet-*SNF6* mutant and promoters bound by Snf6 under yeast-promoting conditions.

To comprehensively elucidate the role of Snf6 in *C. albicans*, its genomic occupancy was determined using chromatin immunoprecipitation coupled to high-density tiling arrays for cells growing in the yeast form in YPD medium. We found that Snf6 bound more than 700 peaks, which corresponded to 418 promoter regions (Table S2). Gene ontology (GO) analysis showed that Snf6 target promoters were strongly enriched for biological processes related to carbohydrate metabolism (Fig. 2A). Snf6 was found to bind both promoters of glycolytic genes (*GLK1*, *PFK1*, *PFK2*, *FBA1*, *CDC19*, *GPM1*, *ENO1*, *TDH3*, *TPI1*, and *HXK2*) and their transcriptional regulators, including Tye7, Gal4, and Ace2 (Table S2). Snf6 targets were also enriched in processes related to host interaction, including oxidative and nitrosative stress response (*TRR1*, *TRX1*, *GPX3*, *CAT1*, *GLX3*, *YHB1*, and *CTA4*), adhesion (*ALS1*), and invasion (*EFG1* and *NRG1*). Other promoter targets were associated with metabolism of both lipids (*MIT1*, *IPT1*, *SCS7*, *LCB4*, *LSP1*, and *OLE1*) and amino acids (*HIS1*, *CYS4*, *ARG4*, *AAT2*, *LEU42*, *GLY1*, *PUT1*, *ILV2*, *MET2*, *GLN1*, *CAR1*, *GCN4*, and *CBF1*), as well as with ammonium transport (*NPR1*, *TPO3*, *FRP3*, and *MEP1*). Taken together, these data show that in a nutrient-rich environment, Snf6 appears to control the nucleosome dynamics of promoters related to sets of different biological processes.

10.1128/mSphere.00497-17.2 TABLE S2  List of gene promoters bound by Snf6 under yeast-promoting conditions (YPD, 30°C) and their GO enrichment analysis. Download TABLE S2, XLSX file, 0.1 MB. Copyright © 2017 Tebbji et al. 2017 Tebbji et al. This content is distributed under the terms of the Creative Commons Attribution 4.0 International license.

### Snf6 is required for both activation and repression of its target genes.

**FIG 3:**
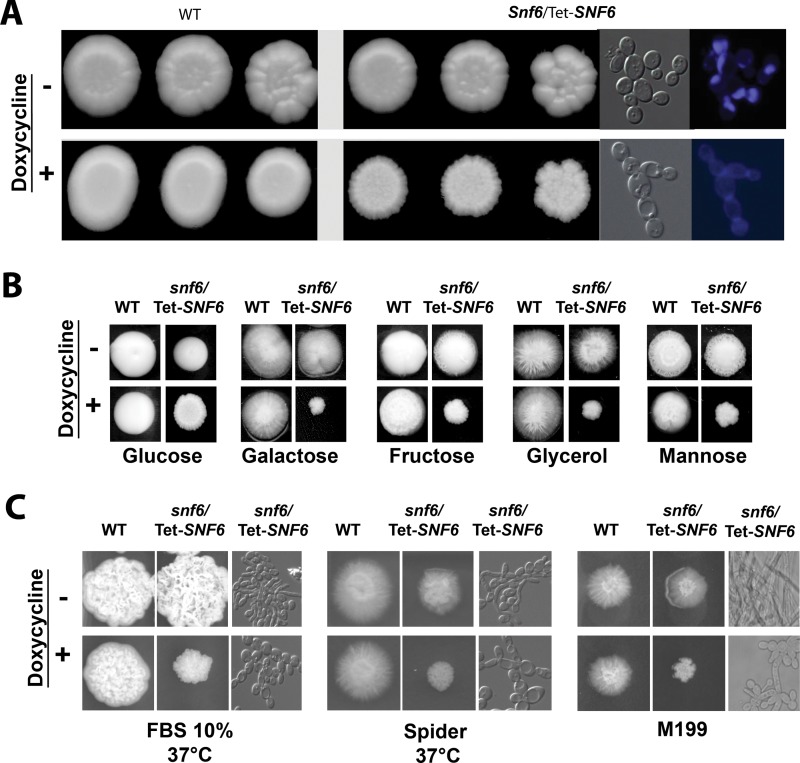
Depletion of *SNF6* led to multiple phenotypes relevant to *C. albicans* fitness and pathogenicity. (A) WT and *snf6*/Tet-SNF6 cells were serially diluted and grown on plates under nonrepressing (YPD) or standard repressing (YPD plus 20 µg/ml doxycycline) conditions for 3 days at 30°C, and the resulting colonies were photographed. Cells from *snf6* mutant colonies were observed by microscopy using differential interference contrast for bright field or through the 4′,6-diamidino-2-phenylindole (DAPI) filter for calcofluor white staining. (B) WT and *snf6*/Tet-SNF6 cells were grown in different solid media with different carbon sources under both repressing (40 µg/ml doxycycline) and nonrepressing conditions. Pictures were taken after 3 days of growth. WT refers to strain CAI4. (C) Snf6 is required for hyphal development. WT and *snf6/*Tet-*SNF6* cells were grown in YPD plus 10% FBS and Spider and M199 media to promote invasive hypha formation in the presence or the absence of doxycycline. Representative pictures were taken after 3 days of growth.

When strains were serially diluted and grown on plates under standard repressing conditions (YPD plus 20 μg/ml doxycycline) for 3 days at 30°C, there was little effect on growth (Fig. 3A). However, the repression of the *SNF6* gene influenced cell and colony morphology; the mutant grew as chains of ellipsoid cells in a budding pattern with branches at 30°C. To relate the genomic occupancy of Snf6 to its role in regulating gene expression, transcriptional profiling of *snf6*/Tet-*SNF6*, the conditional GRACE mutant (16), was performed using microarrays. Snf6-dependent transcripts were identified by comparing the transcriptional profile of *snf6*/Tet-*SNF6* cells treated with doxycycline to that of nontreated *snf6*/Tet-*SNF6* cells. A total of 128 transcripts were differentially regulated, including 57 upregulated and 69 downregulated genes (Table S3). Transcripts that Snf6 failed to repress (upregulated in the mutant) were mainly enriched in functions related to carbohydrate metabolism and stress response (Fig. 2B). Gene ontology (GO) analysis of transcripts that require Snf6 for their activation (downregulated in the mutant) did not give any statistically significant functional enrichment of GO terms. However, a careful analysis of the list of downregulated genes uncovered transcripts associated with cell wall biosynthesis (*SMI1* and C1_14060W_A), glucose transport (*HGT1* and *HGT19*), adhesion (*CSH1*, *SAP9*, and *ALS4*), and vesicle transport (*ALY2*, *VRP1*, and *SEC2*).

10.1128/mSphere.00497-17.3 TABLE S3  List of differentially regulated genes in *snf6*/Tet-*SNF6* mutant under repressible conditions and GO analysis of both up- and downregulated transcripts. Download TABLE S3, XLSX file, 1.6 MB. Copyright © 2017 Tebbji et al. 2017 Tebbji et al. This content is distributed under the terms of the Creative Commons Attribution 4.0 International license.

To further investigate the *C. albicans* cellular pathways whose expression is influenced by inactivation of *SNF6*, we performed gene set enrichment analysis (GSEA). Within the set of genes upregulated in the *snf6* mutant, GSEA detected enrichment for genes downregulated in the yeast *efg1* and *tye7* mutants and genes upregulated in Spider-medium-generated hyphal cells and in Ada2 and Gal4 transcription factor binding (Table S3). In the set of downregulated genes in the *snf6* mutant, the GSEA showed enrichment of SWI4/SWI6 downregulated genes, genes downregulated in Spider-medium-generated hyphal cells, genes suppressed by heat shock, and ribosome genes and a correlation with Tbf1, Fhl1, and Ifh1 transcription factor binding, suggesting that the regulation of the ribosomal protein regulon has been compromised (Table S3).

By overlapping the ChIP-chip data with the list of Snf6 transcriptionally dependent genes, we identified 28 direct Snf6 targets including 15 upregulated and 13 downregulated transcripts (Fig. 2C). These data suggest that Snf6 contributes to both activation and repression of its target genes. Glycolytic genes, in addition to genes for heat shock proteins, were among genes that Snf6 represses directly, while genes that depend on Snf6 for their direct activation belong to diverse metabolic processes (Fig. 2C; Table S3).

### Snf6 is required for carbon utilization, hyphal and invasive growth, and resistance to heat stress.

Since Snf6 binds directly to the promoters of genes related to carbohydrate metabolism and it is required for their proper modulation, we wanted to assess whether it is essential for growth flexibility in media with different carbon sources including fermentable alternative sugars (glucose, fructose, galactose, and mannose) and nonfermentable carbon (glycerol). While no discernible growth defect was observed on glucose, *snf6* strains exhibited a severe growth defect especially when growing in medium with galactose or glycerol as a sole source of carbon (Fig. 3B). This suggests that, as in *S. cerevisiae* (17), Snf6 is required for metabolic flexibility of carbon use.

**FIG 4:**
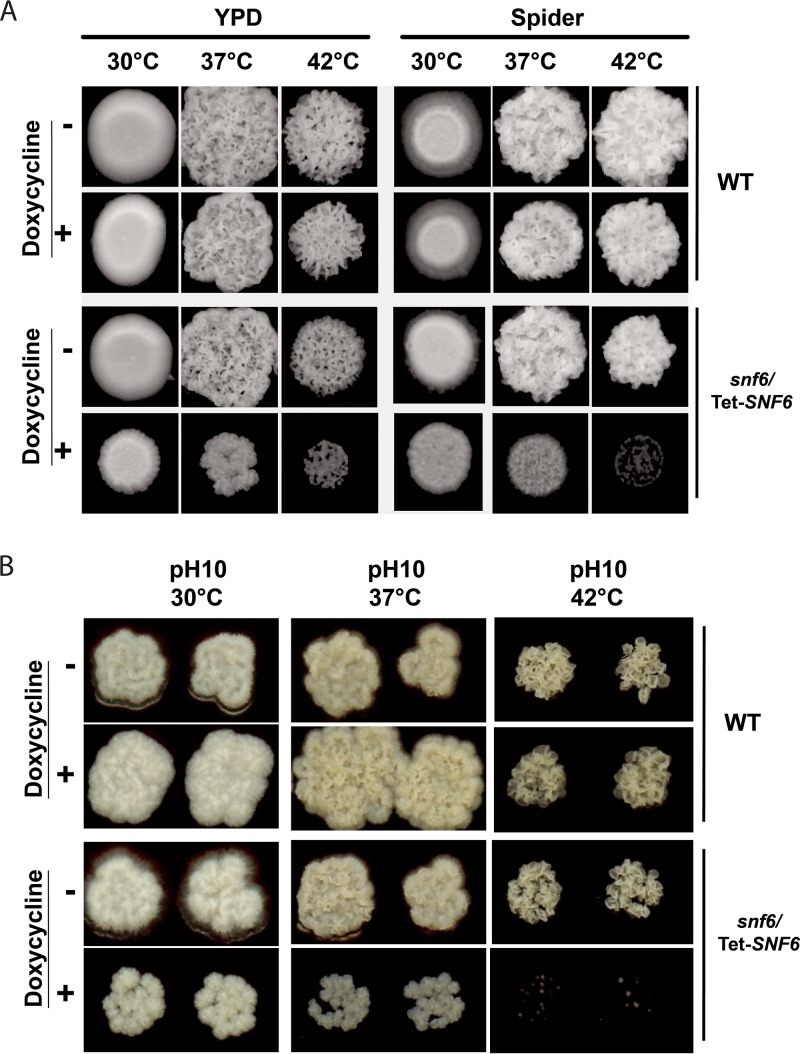
*SNF6* mutant is sensitive to heat stress. (A) Effect of heat stress on *snf6* mutant. Pictures were taken after 3 days of growth at temperatures of 30, 37, and 42°C in YPD and Spider medium. (B) Effect of heat stress on *snf6* mutant under alkaline condition. Pictures were taken after 3 days of growth at temperatures of 30, 37, and 42°C in YPD (pH 10).

Carbon limitation or poor carbon sources are well-known cues that promote the switch of *C. albicans* cells from the yeast to the hyphal state (18). Also, carbohydrate utilization genes are coactivated during the yeast-to-hypha transition (19, 20). Since *snf6* mutant strains exhibited growth defects in different carbon sources, we wanted to test whether this SWI/SNF subunit is required for hyphal formation in response to different cues. The ability of the *snf6* conditional mutant to form hyphae was assessed using fetal bovine serum (FBS) or Spider or M199 medium or alkaline pH. Wild-type (WT) and *snf6/*Tet-*SNF6* strains nontreated with doxycycline formed abundant filaments at the edge of colonies and exhibited a typical wrinkled-colony morphology (Fig. 3C). In contrast, *snf6/*Tet-*SNF6* cells under repressible conditions did not develop invasive filaments, and microscopic analysis of cells from colonies showed swollen yeast cells that form chains (Fig. 3C and 4B).

Since Snf6 also bound directly to heat shock proteins and was required for their activation, we tested the ability of an *snf6* conditional mutant to grow at high temperatures. Our data revealed that, under repressible conditions, *snf6* mutant growth was very sensitive to the high temperature 42°C in contrast to the WT or the *snf6*/Tet-*SNF6* strain without doxycycline treatment (Fig. 4A). When grown at alkaline pH (Fig. 4B), the *snf6* mutant was completely unable to proliferate at the high temperature 42°C compared to the other temperatures of 30°C and 37°C.

### Transcriptional profiling and genome-wide occupancy of Snf6 under hypha-promoting conditions.

**FIG 5:**
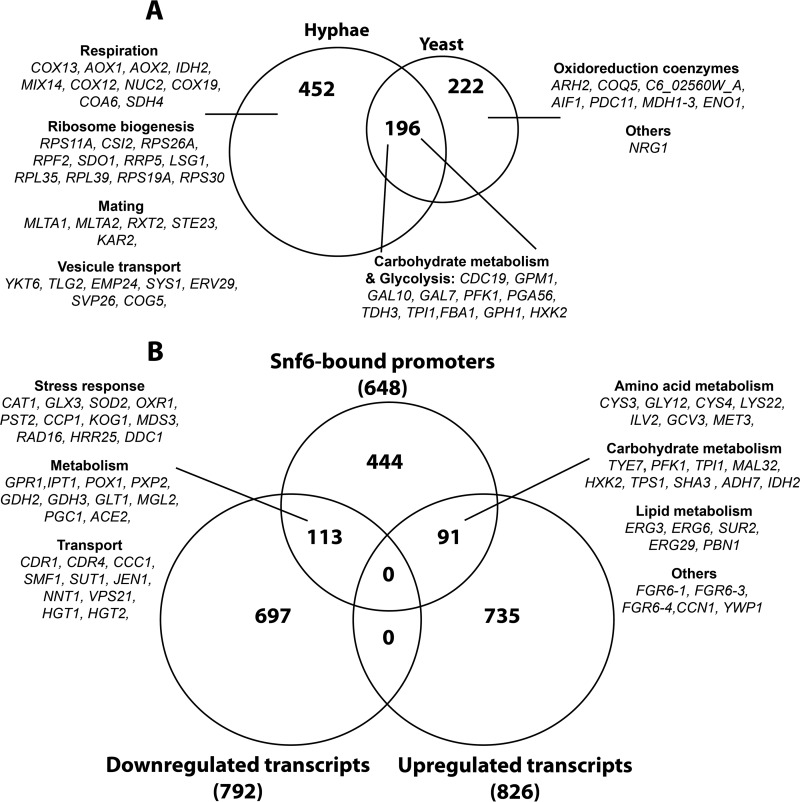
Snf6 regulon under hypha-promoting conditions. (A) Venn diagram showing overlap between Snf6-bound promoters under yeast- and hypha-promoting conditions. (B) Venn diagram showing overlaps between genes differentially regulated in *snf6*/Tet-*SNF6* mutant and promoters bound by Snf6 under hypha-promoting conditions. Relevant functional categories are shown.

Given its critical role in hypha formation, Snf6 might be required to control, at the chromatin level, the transcription of genes that promote morphogenesis in *C. albicans*. To assess this, the genomic occupancy of Snf6 was determined under conditions that stimulate hyphal growth (10% FBS, 37°C). Snf6 bound 648 promoter regions (854 and 814 peaks were detected in both replicates), which correspond to approximately 10% of *C. albicans* genes. A total of 196 promoters occupied by Snf6 were common with the yeast condition and were enriched in genes related to carbohydrate metabolism (Fig. 5A; Table S4). Promoters that were bound specifically under hyphal conditions were associated with different biological processes such as respiration, vesicle transport, mating, and ribosome biogenesis, while those that were unique to the yeast growth condition were mainly enriched in oxidoreduction coenzymes (Fig. 5A). Overall, compared to the yeast growth condition results, these data show that Snf6 expands its binding to include other sets of genes implicated in other biological processes that are involved in the hyphal transcriptional program. Interestingly, Snf6 occupied the promoter of the key transcriptional repressor of hyphal formation, Nrg1, specifically under yeast-promoting conditions.

10.1128/mSphere.00497-17.4 TABLE S4  List of gene promoters bound by Snf6 under hypha-promoting conditions (YPD plus 10% FBS, 37°C) and their GO enrichment analysis. Download TABLE S4, XLSX file, 0.2 MB. Copyright © 2017 Tebbji et al. 2017 Tebbji et al. This content is distributed under the terms of the Creative Commons Attribution 4.0 International license.

Similarly to the yeast growth condition experiments, we used microarray profiling to identify transcripts that depend on Snf6 for their modulation during the yeast-to-hypha transition. We focused our analysis in genes that simultaneously were direct targets of Snf6 and were differentially expressed in the *snf6* conditional mutant under repressible conditions. Snf6 was required to activate genes related to oxidative stress response, drug and hexose transport, and diverse metabolic processes such as lipid and glutamine biosynthesis (Fig. 5B; Table S5). As was seen during yeast-form growth, Snf6 can behave as a repressor of metabolic genes, including carbohydrate, amino acid, and lipid metabolic genes (Fig. 5B). Although Snf6 was essential for hyphal formation, no binding was found at the promoters of hypha-specific genes, such as *HWP1*, *ECE1*, *SOD5*, *ALS3*, and *RBT4*, suggesting that Snf6 or the SWI/SNF complex might control filamentation indirectly through another transcriptional regulator. However, Snf6 binds a large number of members of the FGR (filamentous growth regulator) family (*FGR6*-1, *FGR10*, *FGR6*-3, *FGR3*, *FGR50*, *FGR6*-10, *FGR42*, *FGR51*, and *GPI19*), as well as genes for cell adhesion factors (*ALS1*, *TDH3*, and *DEF1*) and cell separation regulator genes, such as *ACE2* and *ENG1*.

10.1128/mSphere.00497-17.5 TABLE S5  List of differentially regulated genes in *snf6*/Tet-*SNF6* mutant hypha-promoting conditions (YPD plus 10% FBS, 37°C) and GO analysis of both up- and downregulated transcripts. Download TABLE S5, XLSX file, 1.7 MB. Copyright © 2017 Tebbji et al. 2017 Tebbji et al. This content is distributed under the terms of the Creative Commons Attribution 4.0 International license.

GSEAs of the upregulated genes in the mutant under hyphal growth conditions show enrichment in genes downregulated in hyphae and biofilm and a correlation with Tbf1, Fhl1, and Ifh1 transcription factor binding. However, the set of downregulated genes was enriched in genes that were identified to be upregulated during gut intestinal colonization (21), suggesting a potential role of Snf6 in the regulation of gastrointestinal (GI) colonization and dissemination.

## DISCUSSION

This study represents a comprehensive characterization, at the genomic level, of the role of the SWI/SNF complex in the pathogenic yeast *C. albicans*. So far, the *C. albicans* SWI/SNF complex has been implicated in morphogenesis (11) and drug resistance (12) without a focus on its general function on transcriptional regulation at the genome-wide level. We have shown that an SWI/SNF complex subunit, Snf6, was required for both metabolic flexibility and invasive growth, which are key features needed for an opportunistic pathogen such as *C. albicans* to maintain its fitness and pathogenicity inside its host. Snf6 was required for *C. albicans* to utilize different carbon sources, suggesting a pivotal role of this SWI/SNF subunit in carbon metabolic flexibility. Additionally, Snf6 was also required to resist heat stress. Our data suggest a direct transcriptional control of carbohydrate utilization genes as well as heat shock factor genes by Snf6. Surprisingly however, those genes were activated in the *snf6* mutant rather than being downregulated. A similar trend was observed in *S. cerevisiae*, where glycolytic genes were upregulated in an *snf6* (9) or *snf5* (10) mutant. This phenomenon might be explained by the fact that fungal cells could compensate for the loss of SWI/SNF activity by recruiting other chromatin remodelers to mediate the transcriptional activation of carbohydrate or heat shock genes.

Although Snf2, the catalytic subunit of the SWI/SNF complex, and the other core proteins are conserved among eukaryotes, Snf6 is one of the components of the fungal SWI/SNF complex that is not present in the counterpart human complex. Although fungus specific, Snf6 is exclusively present in the hemiascomycetes clade, and even within this phylogenetic group and with the exception of the short region at the N-terminal ends (Fig. 1A), the Snf6 protein sequence is highly divergent. Snf6 protein has no specifically identified functional domain, which suggests that this subunit might play a structural role important for the integrity of the SWI/SNF complex, rather than providing a catalytic function. Indeed, Snf6 was shown to be required for both the structural integrity and DNA-binding activity of the SWI/SNF complex in yeast (10, 22, 23). Recent investigations have shown that the Snf6 subunit interacts with the helicase/SANT-associated (HSA) domain of the catalytic subunit Snf2 (10). Interestingly, this interaction is mediated by the N-terminal conserved region of Snf6 that we have identified here as representing the only conserved part of the protein. The Snf2 HSA domain represents a binding platform for the actin-related proteins Arp7 and Arp9, and it is essential for the chromatin-remodeling activity (24).

Our TAP-MS experiment showed that Snf6 interacts with Sth1, which is the catalytic ATPase subunit of the chromatin-remodeling complex RSC (25). This suggests that Snf6 might be a shared subunit between these two functionally homologous complexes. In the budding yeast, Snf6 is a unique subunit of SWI/SNF, in contrast to Rtt102, Arp7, and Arp9, which are shared with RSC (26, 27). In the fission yeast and metazoans, SWI/SNF and RSC complexes share more than 6 subunits, a much greater degree of overlap than in *S. cerevisiae* (28). An Snf6-Sth1 interaction in *C. albicans* could suggest the possibility that SWI/SNF and RSC complexes might contribute together to chromatin remodeling at common promoters and thus could show functional redundancy. In *S. cerevisiae*, such redundancy was previously reported where RSC and SWI/SNF cooperate with each other in histone eviction at the promoters of heat shock proteins (29).

Similarly, the role of Snf6 in morphogenesis might be mediated through its recruitment by a transcriptional activator or repressor to control the yeast-to-hypha genetic program. Given the consistent number of transcriptional regulators that control this essential virulence trait in *C. albicans*, it is challenging to predict those that might use the SWI/SNF complex. Of note, under hypha-promoting conditions, Snf6 was not found to occupy promoters of key hypha-specific genes, such as *HWP1* and *ECE1*, suggesting that the candidate transcription regulator is an indirect regulator of either those genes or higher-hierarchy regulators of other direct transcriptional regulators. Interestingly, a direct target that Snf6 fails to repress during filamentation was the yeast-specific cell wall protein, Ywp1. This suggests that Snf6 is needed to suppress the morphological yeast-specific transcriptional program when *C. albicans* cells undergo the yeast-to-hypha transition. Furthermore, the Nrg1 promoter was bound exclusively under yeast conditions, which suggests that Snf6 might contribute to the activation of this yeast-promoting transcriptional regulator (30), and when *C. albicans* switches to the filamentous form, Snf6 might release the promoter of Nrg1 to mediate the derepression of hypha-specific genes.

**FIG 6:**
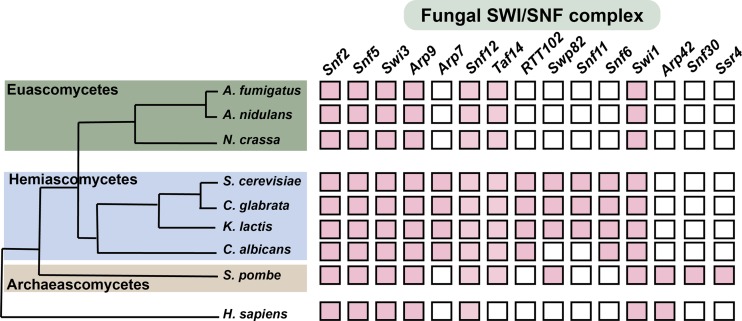
Conservation of fungal SWI/SNF complex subunits across ascomycetes. Different fungal subunits shown were identified from studies in the yeast models *S. cerevisiae* (48) and *S. pombe* (28). Subunits from other fungi were identified based on sequence similarity using Blast analysis. The topology of the phylogenetic tree was inspired by reference 49.

Since Snf6 has no obvious catalytic activity to be used as biochemical readout to screen for such inhibitors, it may be challenging to set up a high-throughput drug discovery routine. In *S. cerevisiae*, Snf6 is required for the maintaining the full structural integrity of SWI/SNF (9, 31). Assuming that this function is conserved in *C. albicans*, an alternative approach to chemically compromise Snf6 function would be identification of small molecules that might dissociate Snf6 from the rest of the SWI/SNF complex. In contrast to Snf6, the fungus-specific SWI/SNF subunits Snf11 and Swp82 were present in the *S. cerevisiae*, *C. glabrata*, and *Kluyveromyces lactis* lineage but absent from the CTG clade and euascomycetes (Fig. 6). Given the role of SWI/SNF in *C. glabrata* biofilm formation (15), chemical dissociation of those subunits could be also be applied to manage infections caused by this resistant growth state. Recently, an increased focus on the druggability of protein-protein interaction has led to identification of potential drugs that reached clinical development (32); however, such an approach has not yet been exploited for antimicrobial discovery. This concept could also be applied to the other fungus-specific SWI/SNF subunits.

We have shown that Snf6 binds directly to gene promoters to mediate both transcriptional activation and repression. This feature is preserved in the SWI/SNF complex of *S. cerevisiae*, *Schizosaccharomyces pombe*, and mammals (14, 28, 33–35). This underlines the point that both transcriptional activators and repressors recruit the SWI/SNF to their target promoters. For instance, in *C. albicans*, carbohydrate and glycolytic genes might be transcriptionally controlled either by activators such as the transcription factors Tye7 and Gal4 (36–38) and Ace2 (39) or by repressors such as Mig1 (40) or Rgt1 (41). All these transcriptional regulators have been shown to play a critical role in host colonization and invasion, and their physical interactions with Snf6 or other subunits of the SWI/SNF complex represent potential therapeutic interfaces to target with small molecules. The feasibility of such an approach has been elegantly demonstrated in *C. glabrata*, where small molecules that disrupt interaction between the drug resistance regulator, Pdr1, and the mediator complex abolished drug resistance in clinical isolates (42). Thus, in addition to targeting protein-protein interactions within the SWI/SNF complex, transcriptional activator/repressor-SWI/SNF interactions represent additional candidates for antifungal therapeutic strategies.

## MATERIALS AND METHODS

### Yeast strains and growth conditions.

Strains used in this study are listed in Table S6 in the supplemental material. For general propagation and maintenance, the strains were cultured at 30°C in yeast extract-peptone-dextrose (YPD) medium supplemented with uridine (2% Bacto peptone, 1% yeast extract, 2% dextrose, and 50 μg/ml uridine, with the addition of 2% agar for solid medium). Cell growth, transformation, and DNA preparation were carried out using standard yeast procedures.

10.1128/mSphere.00497-17.6 TABLE S6  Strains used in this study. Download TABLE S6, DOCX file, 0.02 MB. Copyright © 2017 Tebbji et al. 2017 Tebbji et al. This content is distributed under the terms of the Creative Commons Attribution 4.0 International license.

For gene expression profiling under yeast-promoting conditions, cells were collected directly from agar plates with or without doxycycline (40 µg/ml) after growing for 48 h at 30°C. For the hyphal form, cells were harvested from an agar plate with 10% FBS with or without doxycycline after 48 h at 37°C. Harvested cells were rapidly frozen in liquid nitrogen and immediately processed for RNA extraction.

### *Snf6* GRACE mutant phenotyping.

To promote hyphal growth, *C. albicans* cells were serially diluted and spotted onto a plate of YPD agar containing 10% FBS with or without 40 µg/ml doxycycline. Plates were then incubated for 3 to 4 days at 37°C. To assess the metabolic flexibility of the *snf6* conditional mutant on different carbon sources, cells were plated on YP agar medium containing the appropriate carbon source (glucose, galactose, fructose, mannose, or glycerol) at 2%. Cells were grown at 30°C for 3 days.

### RNA extraction and microarray experiment.

To extract RNA from *C. albicans* cells, samples stored at −80°C were placed on ice, and RNeasy buffer RLT was added to pellets at a buffer/pellet ratio of 10:1 (vol/vol). The pellet was allowed to thaw in the buffer with vortexing briefly at high speed. The resuspended pellet was placed back on ice and divided into 1-ml aliquots in 2-ml screw-cap microcentrifuge tubes containing 0.6 ml of 3-mm-diameter acid-washed glass beads. Samples were homogenized 5 times, for 1 min each, at 4,200 rpm using a BeadBeater homogenizer. Samples were placed on ice for 1 min after each homogenization step. Next, the Qiagen RNeasy protocol was followed as recommended by the supplier. Total RNA samples were eluted in RNase-free H_2_O. RNA quality and integrity were assessed using an Agilent 2100 Bioanalyzer.

cDNA labeling and microarray production were performed as previously described (43). Briefly, 20 μg of total RNA was reverse transcribed using 9 ng of oligo(dT)_21_ and 15 ng of random octamers (Invitrogen) in the presence of Cy3 or Cy5-dCTP (Invitrogen) and 400 U of Superscript III reverse transcriptase (Invitrogen). After cDNA synthesis, template RNA was degraded by adding 2.5 U RNase H (Promega, Madison, WI) and 1 μg RNase A (Pharmacia, Uppsala, Sweden) followed by incubation for 20 min at 37°C. The labeled cDNAs were purified with a QIAquick PCR purification kit (Qiagen). Prior to hybridization, Cy3/Cy5-labeled cDNA was quantified using an ND-1000 UV-visible (UV-Vis) spectrophotometer (NanoDrop, Wilmington, DE) to confirm dye incorporation. DNA microarrays were processed and analyzed as previously described (44).

### Whole-genome location profiling by ChIP-chip and data analysis.

*SNF6* (ORF19.831) was TAP tagged *in vivo* with a TAP-*URA3* PCR product as previously described (45). Transformants were selected on SC-Ura plates, and correct integration of the TAP tag was checked by PCR and Western blotting. The Snf6-TAP fusion was fully functional since deleting the nontagged allele in this strain revealed phenotypes comparable to the parental strain (SN148) under all growth conditions investigated in the current work (data not shown). Cells were grown to an optical density at 600 nm (OD_600_) of 2 in 40 ml of YPD. The subsequent steps of DNA cross-linking, DNA shearing, chromatin immunoprecipitation, and DNA labeling with Cy dyes were conducted exactly as described by Lavoie et al. (45). Tiling arrays were cohybridized with tagged immunoprecipitated (Cy5-labeled) and mock-immunoprecipitated (untagged SN148 strain; Cy3-labeled) DNA samples. Microarray hybridization, washing, and scanning were performed as described by Sellam et al. (44). The significance cutoff was determined using the distribution of log ratios for each factor. It was set at 2 standard deviations from the mean of log-transformed fold enrichments. Values shown are of two biological replicates derived from independently isolated transformants of tagged and mock constructs. Peak detection was performed using Gaussian edge detection applied to the smoothed probe signal curve as previously described (46).

Tiling arrays were designed as previously described (47). Briefly, starting from sequences from the *C. albicans* Genome Assembly 21 and the *MTL* alpha locus, we extracted a continuous series of 242,860 60-bp oligonucleotides, each overlapping by 1 bp. We then eliminated 2,062 probes containing stretches of 13 or more A or T nucleotides. The remaining 240,798 sequences were then used to produce sense (Watson strand) and antisense (Crick strand) whole-genome tiling arrays using the Agilent Technologies eArray platform.

### Immunoblotting and coimmunoprecipitation.

*C. albicans* Snf6-TAP and other double-tagged strains (Table S6) were grown to mid-log phase in YPD medium. Cells at a final OD_600_ of 1 to 1.5 were harvested by centrifugation and lysed by bead beating in IP150 buffer (50 mM Tris-HCl [pH 7.4], 150 mM NaCl, 2 mM MgCl_2_, 0.1% Nonidet P-40) supplemented with a Complete Mini protease inhibitor mixture tablet (Roche Applied Science) and 1 mM phenylmethylsulfonyl fluoride (PMSF). The lysates were then cleared by centrifugation, and protein concentration was estimated using the Bradford assay. One milligram of total protein was added to 50 µl of anti-tap IgG Sepharose beads (GE), monoclonal mouse anti-Myc (9E10), or antihemagglutinin (anti-HA) (12CA5) (Roche Applied Science) and incubated at 4°C with end-over-end mixing overnight. The next morning, beads were centrifuged at 2,000 rpm at 4°C, washed three times with IP150 buffer, boiled with SDS-PAGE loading buffer, and resolved by 4 to 20% gradient SDS-PAGE. Proteins were transferred onto a nitrocellulose membrane and analyzed by Western blotting using rabbit anti-TAP polyclonal antibody (1:2,500) (GenScript), rabbit anti-Myc (1:1,000) (Santa Cruz Biotechnology), or anti-HA (1:2,500) (Roche Applied Science) wherever applicable.

### TAPs and liquid chromatography-tandem mass spectrometry (LC-MS/MS).

To assess protein-protein interactions on a large scale, we used an Snf6 TAP tag construct. Tandem affinity purifications were performed as described at the website http://depts.washington.edu/yeastrc/pages/plasmids.html and then precipitated with trichloroacetic acid (TCA) as described by Tebbji et al. (47). The mass spectrometer used was the Velos LTQ-Orbitrap (Thermo Fisher, San Jose, CA). Raw mass spectrometric data were processed using Proteome Discoverer 1.3. Spectra were searched against a *C. albicans* SC5314 database obtained from http://www.candidagenome.org containing 6,215 protein sequence entries.

### Data availability.

Raw and processed microarray and ChIP-chip data have been submitted to the GEO database under accession no. GSE106278. The averages of all gene expression of the *snf6* mutant in yeast and hyphae are provided in Supplemental Tables S3 and S5 (sheets named “All genes value”). All raw data regarding the peaks of Snf6 binding are in Supplemental Tables S2 and S4 (sheets named “Snf6 peaks”).
