# Supplementary material for: The Genomic Landscape of the Fungus-Specific SWI/SNF Complex Subunit, Snf6, in Candida albicans
Source: mSphere. 2017 Nov 15;2(6):e00497-17. doi: 10.1128/mSphere.00497-17 (PMC5687922; doi:10.1128/mSphere.00497-17)
Supplement: TABLE S6 [file sph006172405st6.docx]

Table S6

| Strain | Parental strain | Genotype or description | Reference |
| --- | --- | --- | --- |
| SC5314  SN148  Cass1  Snf6 GRACE  Snf6-tap  Snf6 Myc/Snf2 HA  Snf6 Myc/Swi1 HA | SN76  CAI4  CAI4  SN148  SN148  SN148 | *Clinical isolate*  *arg4Δ/arg4Δ leu2Δ/leu2Δ his1Δ/his1Δ*  *ura3Δ::imm434/ura3Δ::imm434*  *iro1Δ::imm434/iro1Δ::imm434*  *his3::hisG/his3::hisG leu2::tetRGAL4AD-URA3/LEU2*  *snf6::his3::hisG/his3::hisG leu2::tetRGAL4AD-URA3/LEU2*  *SNF6/SNF6-TAP-URA3*    *SNIF6/SNIF6-Myc-URA3 : SNF2/SNF2- HA-His1*  *SNIF6/SNIF6-Myc-URA3 : SWI1/SWI1- HA-His1* | [1]  [2]  [3]  [4]  This study  This study  This study |

1. Gillum A, Tsay E, Kirsch D (1984) Isolation of the Candida albicans gene for orotidine-5′-phosphate decarboxylase by complementation of S. cerevisiae ura3 and E. coli pyrF mutations - Springer. Molecular and General Genetics MGG.

2. Noble SM, Johnson AD (2005) Strains and Strategies for Large-Scale Gene Deletion Studies of the Diploid Human Fungal Pathogen Candida albicans. Eukaryotic Cell 4: 298–309. doi:10.1128/EC.4.2.298-309.2005.

3. Fonzi WA, Irwin MY (1993) Isogenic strain construction and gene mapping in Candida albicans. Genetics 134: 717–728.

4. Roemer T, Jiang B, Davison J, Ketela T, Veillette K, et al. (2003) Large-scale essential gene identification in Candida albicans and applications to antifungal drug discovery. Mol Microbiol 50: 167–181.
